# Identification of long non-coding RNA competing interactions and biological pathways associated with prognosis in pediatric and adolescent cytogenetically normal acute myeloid leukemia

**DOI:** 10.1186/s12935-018-0621-0

**Published:** 2018-08-28

**Authors:** Xuejiao Yin, Sui Huang, Ruiqi Zhu, Fengjuan Fan, Chunyan Sun, Yu Hu

**Affiliations:** 1 Institute of Hematology, Union Hospital, Tongji Medical College, Huazhong University of Science and Technology, Wuhan, 430022 China; 2 Collaborative Innovation Center of Hematology, Huazhong University of Science and Technology, Wuhan, 430022 China

**Keywords:** Pediatric and adolescent, CN-AML, LncRNA, ceRNA network, WGCNA, Biological pathways, Prognosis

## Abstract

**Background:**

LncRNAs can regulate miRNAs and mRNAs by sequestering and binding them. Indeed, many researchers have reported lncRNA mediated-competing endogenous RNAs (ceRNAs) could regulate the progression of solid tumors. However, the roles of ceRNA in acute myeloid leukemia (AML), especially in pediatric and adolescent AML, were not completely expounded.

**Materials and methods:**

27 cytogenetically normal acute myeloid leukemia (CN-AML) patients under 18 years old with corresponding clinical data were selected from the cancer genome atlas (TCGA), which was a large sample sequencing database of RNA sequencing. We constructed a survival specific ceRNA network, and investigated its associations with patients’ clinical information by analyzing the data from TCGA.

**Results:**

We identified survival specific lncRNAs, miRNAs and mRNAs, and constructed a survival specific ceRNA network of CN-AML patients and a weighted correlation network. Furthermore, we identified 4 biological pathways associated with OS and selected the most enriched pathway ‘Transcriptional misregulation in cancer’ to verify that it could accurately predict younger CN-AML patients’ prognosis to guide treatment.

**Conclusions:**

We successfully constructed a survival specific ceRNA network which could provide a new approach to lncRNA research in younger CN-AML. Importantly, we constructed a weighted correlation network to overcome the difficulty in biological interpretation of individual genes.

**Electronic supplementary material:**

The online version of this article (10.1186/s12935-018-0621-0) contains supplementary material, which is available to authorized users.

## Background

Long non-coding RNAs (lncRNA) were a group of noncoding RNAs with more than 200 bp, which has intrinsic advantages over the use of protein-coding RNAs in diagnostics [1]. LncRNAs play critical roles in diverse biological functions, including nuclear architecture, regulation of gene expression, immune surveillance, cancer development and maintenance of tumorigenesis. LncRNAs can regulate miRNAs and mRNAs by sequestering and binding them, and competing endogenous RNA (ceRNA) mechanism attracted more and more attention since it was firstly proposed by Salmena et al. [2]. CeRNA is a complex post-transcriptional regulatory network using miRNA response elements (MREs) to compete for the binding of miRNAs thereby implementing mutual control between mRNAs, lncRNAs and miRNA [3]. A lot of studies have reported that ceRNA mechanism plays critical roles in solid tumor progression [4–6]. Junge et al. [7], Ding et al. [8] and Chen et al. [9] also reported highly-upregulated RUNX1T1, C-Myc and CCAT1 acts as competing endogenous RNA (ceRNA) in adult acute myeloid leukemia. However, little has been done in pediatric and adolescent cytogenetically normal acute myeloid leukemia (CN-AML) patients. Although board overlap lies between AML adults and pediatric patients in pathogenesis, diagnosis, treatment and prognosis of the diseases, differences still exist.

CN-AML, a most common AML type, is characterized by the absence of microscopically detectable chromosome abnormalities, but mutations, epigenetic changes and dysregulated expression signatures exists, respectively [10]. Some reports have shown CN-AML patients harboring mutations including NPM1, CEBPA, FLT3-ITD and WT1 were associated with an disparate prognosis [11]. With the advent of high-throughput technologies such as microarray and next-generation sequencing, some prognostic gene expression signatures have been proposed. These findings helped focus targeted therapies for CN-AML with significantly heterogeneous outcomes [12]. The genes in a prognostic gene signature were considered as independent individuals, which may result in ignoring potential relationships between the genes [13]. To overcome these challenges and to reduce the difficulty in biological interpretation, we constructed a weighted correlation network and the significant modules associated with prognosis were selected [14]. Based on these modules information, we identified 4 biological pathways associated with overall survival (OS). Next the most enriched pathway was selected to verify whether it could accurately predict pediatric and adolescent CN-AML patients’ prognosis and guide treatment. To our knowledge, our study is the first to construct survival specific ceRNAs co-expression network and identify survival specific biological pathways in pediatric and adolescent CN-AML patients. This approach can help to clarify the functions of lncRNAs in younger CN-AML patients.

## Materials and methods

### TCGA AML dataset

A total of 27 patients with AML were collected from the cancer genome atlas (TCGA) database. The criteria of exclusion were set as follows: (i) patients were not cytogenetically normal or cytogenetically information unknown; (ii) patients didn’t have lncRNA, mRNA, miRNA information; (iii) patients were alive and the last contact days were unavailable. Overall, 27 CN-AML patients who were under 18 at diagnosis were included in our study. This study was fully compliance with the publication guidelines provided by TCGA. The data were obtained from TCGA, so the approval of ethics committee was not needed.

### Data processing

TCGA database provided the normalized count data of RNA sequencing including lncRNA and mRNA expression profiles by RNASeqV2 system. The STAD level 3 microRNA sequencing (miRNAseq) data, downloaded from TCGA database, were collected by Illumina HiSeq 2000 microRNA sequencing platforms. TCGA database have already normalized these RNA expression profile data, so no further normalization was required. In the next step, each lncRNA, miRNA and mRNA were respectively put into univariate cox’s model. P value < 0.05 as screening criteria were used to select lncRNA, miRNA and mRNA which were significantly associated with OS. Clinical information including age at diagnosis, gender, wbc at diagnosis, bone marrow blasts, peripheral blasts and molecular mutations (FLT3-ITD, NPM1, CEBPA) were also used to build univariate cox’s model under the same standard. To explore the relationship between prognosis and the key lncRNAs, miRNAs and mRNAs involved in the ceRNA network, Kaplan–Meier curve were carried out at a P value < 0.05. Each significant mRNA identified by univariate cox was further chosen to construct a weighted correlation network and the significant modules associated with prognosis were selected for further exploration. The mRNAs in the specific modules associated with OS were further implemented by functional enrichment analysis. The most enriched pathway was selected by carrying out multivariate cox regression formula. Prognostic models were reconstructed based on the relative contributions of each of the genes in the cox analysis, as described in the following equation: The risk score = Z1G1 + Z2G2 + Z3G3 + ……ZnGn (z is z scores obtained from the Cox analysis and G is the expression value of each gene). Then, the patients were classified into 2 groups (high risk vs low risk) based on the median value of the predictor score. Survival times were compared between the 2 groups by using the Kaplan–Meier analysis at a P value < 0.05.

### Construction of the ceRNA network

We used miRanda (http://www.microrna.org/microrna/home.do) to find the lncRNA–miRNA interactions, and using miRBase targets (http://mirdb.org/miRDB/), miRTarBase (http://mirtarbase.mbc.nctu.edu.tw/) and Targetscan (http://www.targetscan.org/) to predict target genes. Predicted targets are ranked according to the predicted efficacy of targeting as calculated using cumulative weighted context ++ scores of the sites, and scores < − 0.4 were selected as target genes of each miRNA. Then, according to the theory of ceRNA, we chose the miRNA negatively regulated intersection expression of lncRNAs and mRNAs to construct the ceRNA network. The ceRNA networks were constructed and visualized using Cytoscape v3.0.

### Weighted correlation network construction

Gene correlation is described as a network in which the relationship between the connected genes is represented by the weight. Each gene is described as a node. The edge weight between the connected nodes is the pairwise pearson coefficient. In gene correlation network construction, an adjacency matrix and an adjacency function are defined. Using adjacency function the co-expression similarity between genes can be described as connection strength. The node dissimilarity is input to hierarchical clustering to define network modules. From the clustering tree many gene co-expression modules are discovered. In the construction of hierarchical clustering tree, a dynamic shear algorithm based on tree branch shape is used. R Bioconductor was performed to construct a weighted correlation network.

### GO, pathway analysis, PPI network establishment and leave-one-out cross validation (LOO-CV)

The Database for Annotation, Visualization and Integrated Discovery (DAVID, http://david.abcc.ncifcrf.gov/) was used to perform Gene Ontology (GO) and Kyoto Encyclopedia of Genes and Genomes (KEGG) pathway analysis. Protein–protein interactions (PPI) network of survival specific genes was constructed using Search Tool for the Retrieval of Interacting Genes/Proteins (STRING, http://string.embl.de/). In the network, genes represent nodes and the interactions between the nodes represent edges. The important nodes with high degree in the network were obtained, namely hub nodes. The co-expresses value > 0.4 was used as the cutoff criterion. R Bioconductor was used to perform leave-one-out cross validation.

## Results

### Survival specific lncRNAs, miRNAs and mRNAs in cytogenetically normal AML and ceRNA network construction

After univariate cox’s model filtering of the data extracted from TCGA database, we identified 1402 lncRNAs, 56 miRNA, and 2232 mRNA negatively associated with overall survival, as well as 34 lncRNAs, 46 miRNA, and 54 mRNA positively associated with overall survival (P < 0.05). Next, based on the theory that lncRNA can regulate miRNA abundance by sequestering and binding them, acting as so-called miRNA sponges, we intended to establish the lncRNA–miRNA–mRNA ceRNAs network. 7 miRNAs targeted 47 key lncRNAs were predicted though miRcode, and 56 target genes of these 7 miRNAs were predicted by three prediction tools—TargetScan, miRanda and miRTarBase. Considering false positive rate of prediction tools, predicted targets are ranked according to the predicted efficiency of targeting as calculated using cumulative weighted context ++ scores of the sites. According to the theory of ceRNA, we then chose the miRNA that negatively regulated intersection expression of lncRNAs and mRNAs to construct the ceRNA network using Cytoscape 3.0 (Table 1).

**Table 1:** miRNAs targeted specifc intersection key lncRNAs and mRNAs in ceRNA network

miRNAs	*lncRNAs*	*mRNAs*
hsa-mir-25	TTTY9B, AC068020.1, C11orf44, DLEU1, CASC2, AC024563.1, AL356475.1, AC083799.1, AC006305.1, POLR2J4, AC009495.1, LINC00365, LINC00502, LINC00392, MIR4500HG, AC104445.1, CRNDE, LINC00504, C8orf49, VENTXP1	RSBN1, CNIH1, TWF1, MFF, CCSER2, BAZ2B, SMU1, PTEN, SLC25A32, FOXN2, RRN3, DDX3X, EDEM1, NUFIP2, ZFC3H1, TMF1, KLHL15, VPS4B, CNEP1R1, DNAJB9, SLC25A36, GOLGA8J, SOX11, KIF5B, KIAA1109, PTAR1, SSFA2, GPBP1L1
hsa-mir-363	AC011498.1, AC092811.1, AC108134.2	
hsa-mir-506	AC108134.2	
hsa-mir-551a	LINC00470, DLEU1, AC079949.1	
hsa-mir-221	C15orf54, CASC2, LINC00158, AC011467.1, AC006305.1, LINC00326, BPESC1, LINC00460, AL356489.1, CRNDE, C5orf17	PCDHA4, CHORDC1, RAB1A, HMBOX1, PPP6C, BRWD1, FBXO28, SNX4, FMR1, UBE2J1, PCDHA6, PCDHA5, ZKSCAN8, PAIP2
hsa-mir-100	DLEU1, AC105206.1, POLR2J4, LINC00365, C8orf49	KBTBD8, SMARCA5
hsa-mir-204	AC092811.1, AC068831.1	

**Fig. 1:**
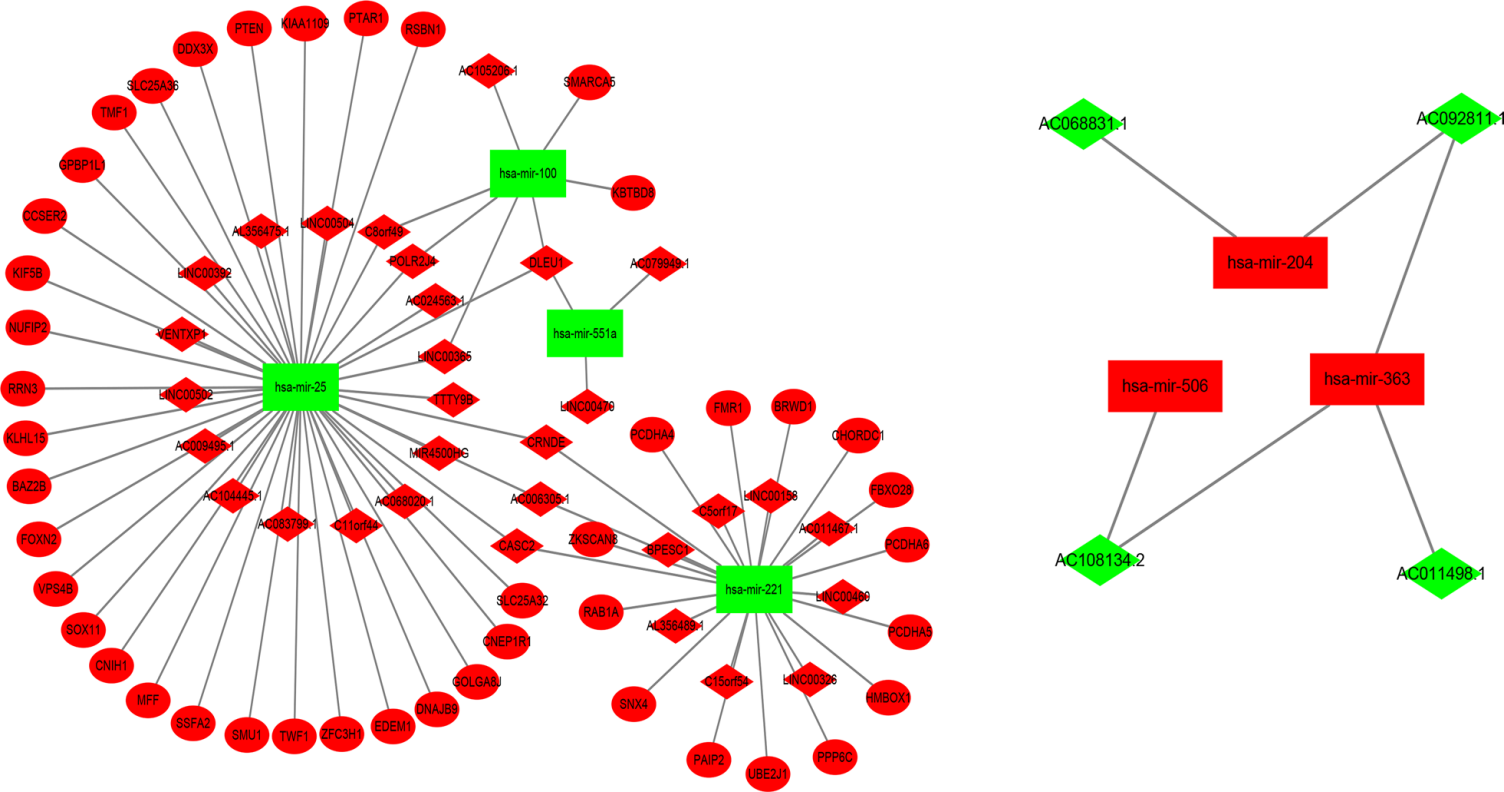
The lncRNA–miRNA–mRNA ceRNA network. Red rhombus: upregulated lncRNAs, red ellipse: upregulated mRNAs, and red rectangle: upregulated miRNAs. Green rhombus: downregulated lncRNAs, and green rectangle: downregulated miRNAs

Finally, 35 lncRNAs, 7 miRNAs and 44 mRNAs were involved in the ceRNA network (Fig. 1). Furthermore, to identify their association with prognostic characteristics, the Kaplan–Meier curve was applied. 6 lncRNAs, 1 miRNA and 14 mRNAs were found significantly associated with cytogenetically normal AML patients’ overall survival (log-rank P < 0.05) (Additional file 1: Figure S1; Additional file 2: Figure S2; Additional file 3: Figure S3). Among these significant RNAs, 4 lncRNAs (AL356475.1, CRNDE, LINC00158 and LINC00504), 1 miRNA (hsa-mir-363) and all 14 mRNAs (BRWD1, CNIH1, FMR1, GOLGA8J, GPBP1L1, HMBOX1, KIF5B, NUFIP2, PTAR1, RRN3, SNX4, TMF1, ZFC3H1 and ZKSCAN8) were negatively associated with overall survival (P < 0.05), while the remaining 2 lncRNA (AC011498.1 and AC092811.1) were positively associated with overall survival (P < 0.05).

### Correlation of lncRNAs and miRNAs signature with clinical or laboratory features and gene alterations

The 35 key lncRNAs and 7 miRNAs in the ceRNA network were further analyzed for the association with the clinical features, such as age at diagnosis, gender, WBC at diagnosis, bone marrow blasts, peripheral blasts and molecular mutations (FLT3-ITD, NPM1, CEBPA) (Additional file 4: Table S1). We found that CRNDE, LINC00504 and hsa-mir-363 were associated with peripheral blasts, CRNDE were associated with bone marrow blasts, CASC2 and CRNDE were associated with WBC at diagnosis. However, there was no association of other lncRNA, miRNA with any clinical features. In addition, we found patients with high level of AC011498.1 were more likely to carry NPM1 mutation and CEBPA mutation compared with patients with low level of the lncRNA. Moreover, patients with high levels of CRNED,LINC00504 and hsa-mir-363 were carried CEBPA mutation. Association between miRNAs, race and ethnicity were also assessed (Additional file 5: Table S3). However, no links were found among miRNA, race and and ethnicity.

### Co-expression module establishment

**Fig. 2:**
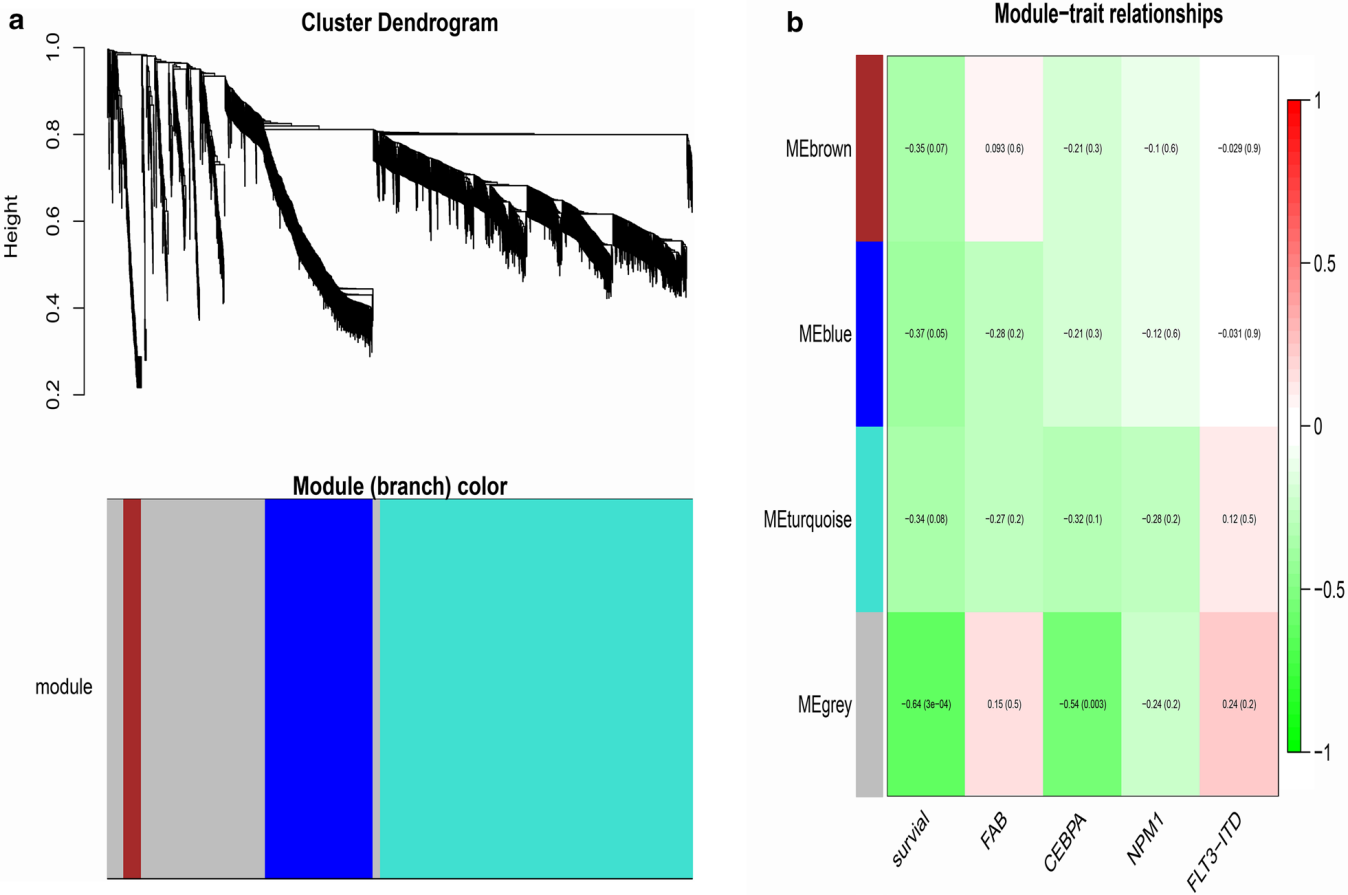
**a** Gene co-expression modules in CN-AML. Clustering dendrogram of genes, with dissimilarity based on topological overlap, together with assigned module colors. **b** The relationship between co-expression modules and Clinical characteristics in CN-AML. Previous digit shows module coefficient and the numbers in brackets represent P value

The 2286 survival specific mRNAs were further chosen for co-expression exploring and a weighted correlation network was constructed. Weighted gene co-expression network analysis is a systems biology method for describing the correlation patterns among genes across microarray samples and exploring the hiding and biologic patterns Fig. 2a showed hierarchical clustering of these 2286 mRNAs and the corresponding gene co-expression module. The color bars correspond to four gene co-expression modules including blue, brown, grey and turquoise modules. The gene number in these modules is 421, 69, 577 and 1218 respectively. To further identify co-expression modules associated with disease progression, clinical characteristics of the patients including survival time, FAB category and molecular alteration associated with prognosis (FLT3-ITD, NPM1, CEBPA) were explored Fig. 2b showed the module significance. Grey module were positively associated with the cytogenetically normal AML patients’ overall survival (cor = 0.64, P < 0.05) and CEBPA mutation (cor = 0.54, P < 0.05).

### Functional enrichment analysis and establishment PPI network of survival specific genes

**Fig. 3:**
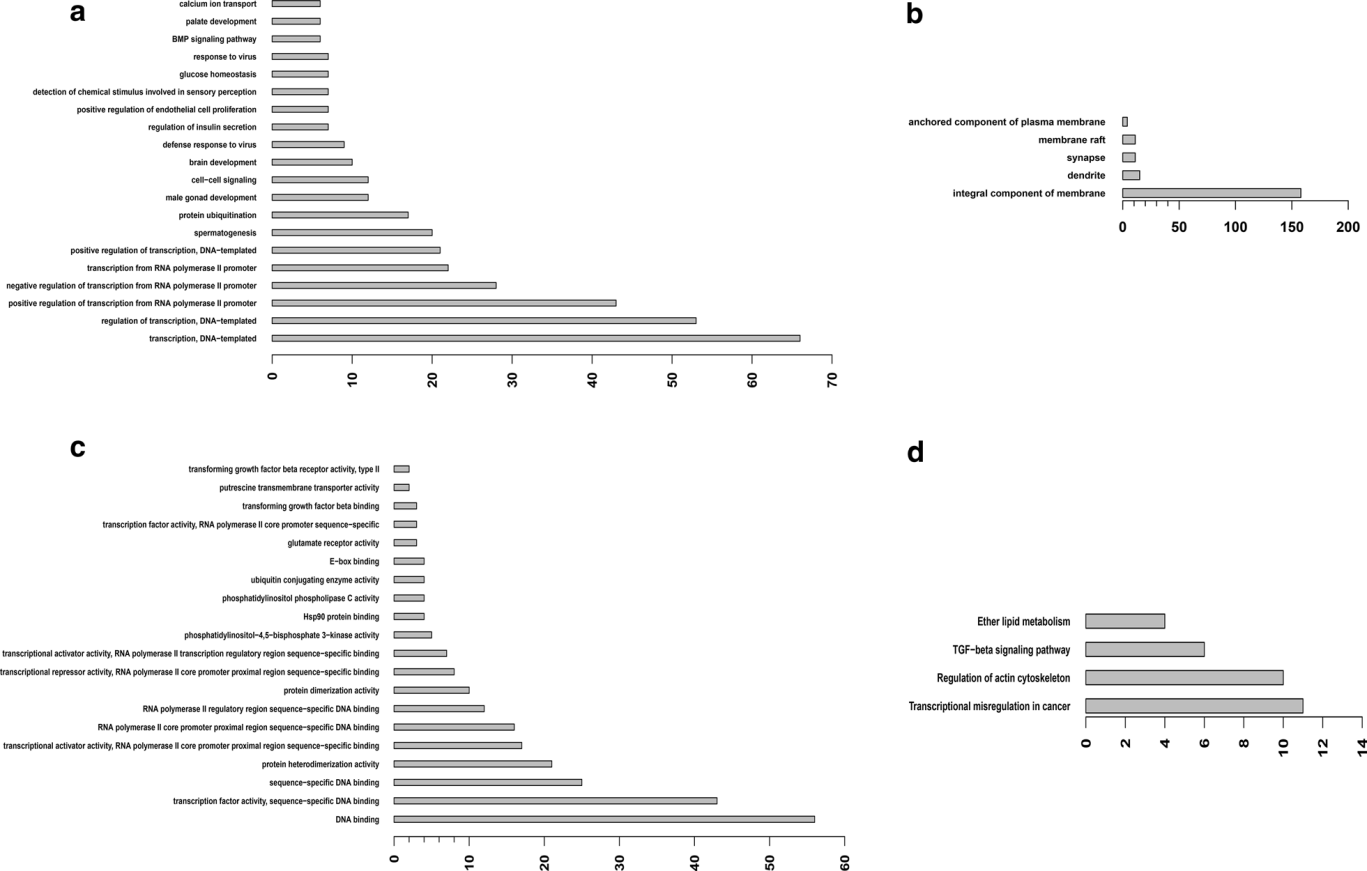
Top 20 enrichment of GO terms and pathways for mRNAs in grey module (the bar plot shows the enrichment scores of the significant enrichment GO terms and pathways) **a** biological process (GO:BP); **b** cellular component (GO:CC); **c** molecular function (GO:MF); **d** pathways(KEGG)

To provide more information for predicting prognosis and guiding treatment in younger CN-AML patients, mRNAs in grey module were applied to select biological pathways associated with OS using gene ontology (GO) enrichment analysis and KEGG pathway analysis. GO analysis showed that the most significantly enriched GO terms were “transcription, DNA-templated” (ontology: BP), “integral component of membrane” (ontology: CC) and “DNA binding” (ontology: MF) (Fig. 3). Enrichment provides a measure of the significance of the function, and as the enrichment increases, the corresponding function is more specific.

Furthermore, KEGG pathway analysis showed that 4 pathways associated with OS and the most enriched pathway was ‘Transcriptional misregulation in cancer’ (Fig. 3). Among these pathways, the ‘Transcriptional misregulation in cancer’ pathway was associated with myeloid leukemogenesis and AML cell functions [15], the ‘TGF-beta signaling pathway’ was involved in the carcinogenesis [16], the ‘Regulation of actin cytoskeleton’ has been investigated as a cause of tumor invasion and metastasis [17] and ‘Ether lipid metabolism’ was involved in the development of cancer [18].

**Fig. 4:**
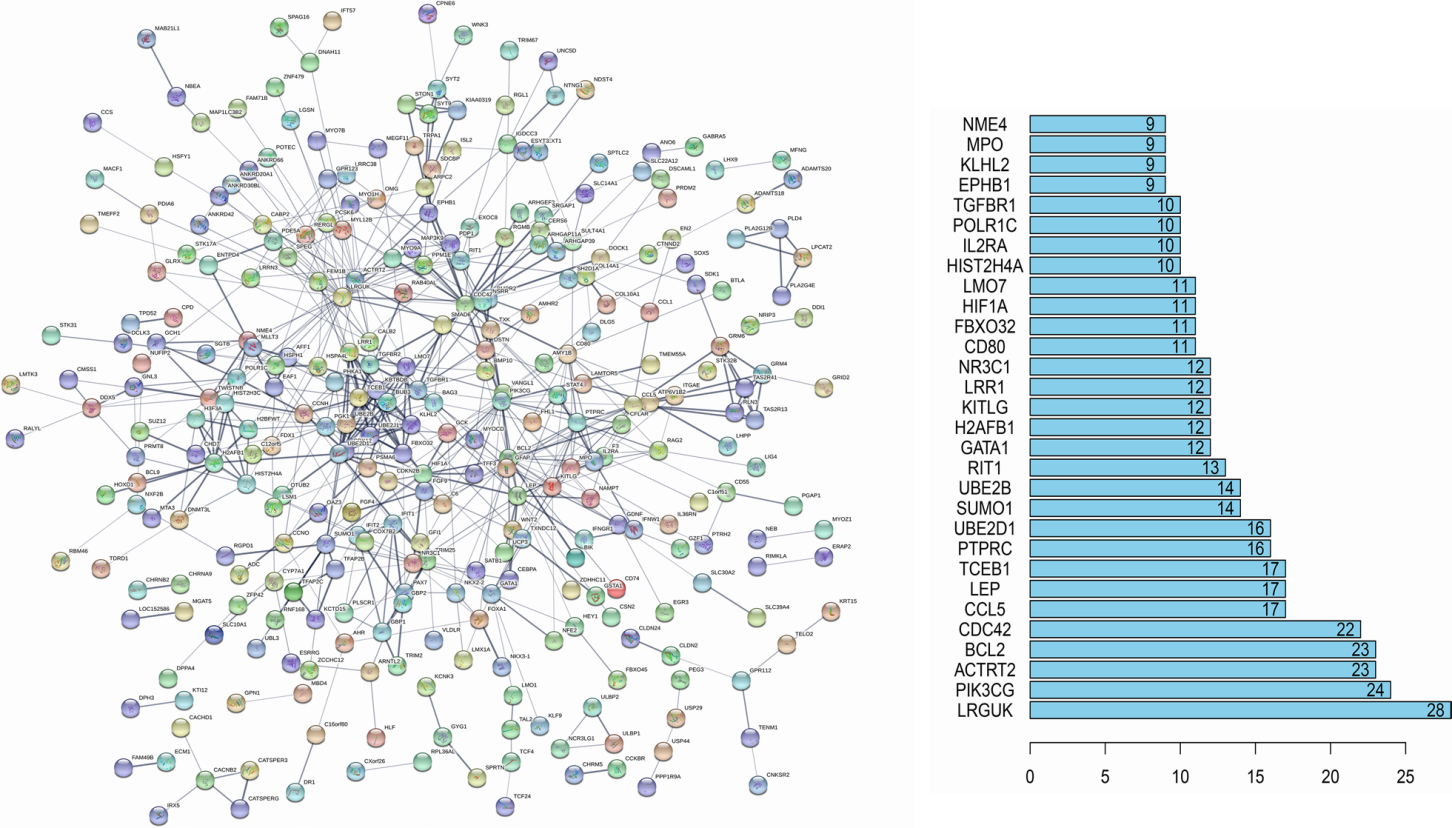
Protein–protein interaction network of genes in grey module and Hub genes selected from protein–protein interaction network (the bar plot shows the enrichment scores of the interactions between the nodes)

The protein–protein interactions (PPI) network of survival specific genes in grey module was next constructed by STRING, which was composed of 312 nodes and 601 edges (Fig. 4). Moreover, 30 genes in the PPI network were identified as hub genes in cytogenetically normal AML, including BCL2 and HIF1A genes, when “Degrees ≥ 9” was set as the cut-off criterion.

### Construction and performance of prognostic pathway-based signatures

**Fig. 5:**
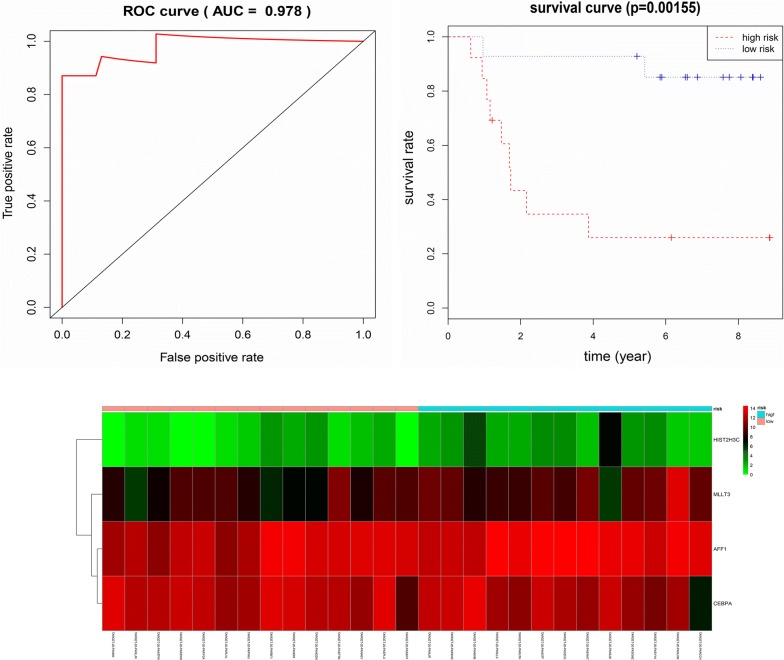
Kaplan–Meier for OS in low risk and high risk groups. AUC curve for the risk score, heatmap for 4 mRNAs expression level and survival status in all 27 patients

Next, the most enriched network ‘Transcriptional misregulation in cancer’ was carried out multivariate cox to verify whether it could accurately predict pediatric and adolescent CN-AML patients’ prognosis. After multivariate cox’s model filtering, we identified 4 mRNAs (MLLT3, CEBPA, HIST2H3C and AFF1) in ‘Transcriptional misregulation in cancer’ pathway were independently associated with prognosis. Only CEBPA were positively associated with OS, the remaining 3 mRNAs were all negatively associated with OS. The risk score was calculated through the four mRNAs status and their weight on OS, which is represented by the β coefficient in multivariate cox model. The risk score = (0.670 * status of MLLT3) − (0.749 * status of CEBPA) + (0.962 * status of HIST2H3C) + (0.824 * status of AFF1). The patients were then classified into 2 groups (high risk vs low risk) based on the median value of the predictor score and the Kaplan–Meier curve was applied. As shown in Fig. 5, the survival time between the 2 groups (high risk vs low risk), created based on the prognostic pathways, was significantly different (P < 0.05) (Fig. 5). The 3-year-Area Under Curve (AUC) of signature was 0.978 (Fig. 5). The heatmap were also created to evaluate the signature, and the results distinctly demonstrated that most dead patients were in high risk group and had worse OS (Fig. 5). Therefore, our 4 mRNA signatures (MLLT3, CEBPA, HIST2H3C and AFF1) may offer an approach for risk assessment and predicting the prognosis in pediatric and adolescent CN-AML patients.

### Comparison of ‘Transcriptional misregulation in cancer’ pathway with other prognostic parameters and leave-one-out cross validation signature

Univariate Cox analysis was used to test clinical parameters with the prognosis. The results showed gender, bone marrow blasts and peripheral blasts could not predict the outcome, while age at diagnosis, FLT3-ITD and WT1 mutations were significantly associated with survival (Additional file 6: Table S2). After adjusting for age at diagnosis, FLT3-ITD and WT1 mutations, our prognostic signatures remained as independent prognostic factors in the multivariate analysis (P < 0.05).

Leave-one-out cross validation test (LOO-CV) is powerful in estimating a model’s performance. To verify whether the 4-mRNA signature could predict other pediatric and adolescent CN-AML patients’ prognosis, LOO-CV was applied. The results showed the AUC of signature was 0.604 which validated the 4-mRNA signature performed well and could successfully predict the prognosis of pediatric and adolescent CN-AML patients.

## Discussion

Pediatric and adolescent acute myeloid leukemia (AML) with incidence of approximately 7 occurrences per 1 million children annually is a rare type of childhood cancer [19]. Because high incidence of severe and dose-limiting short- and long-term toxicities happened, younger AML therapy was a big challenge for patients, their families, and care providers [20]. Although multiple national and international cooperative groups have contributed to an evolving treatment strategy including four to five kinds of intensive myelosuppressive chemotherapy and stem cell transplantation over several decades, there was only a mild decline in younger AML mortality. Do¨hner et al. [21] has been reported State-of-the-art recommendations in adult AML. But little has been done in pediatric and adolescent AML patients. Although there were broad overlap in diagnosis, treatment and prognosis for AML, differences still exist between adolescent and adult patients [11]. Hence, to improve younger AML’s prognosis, the pivotal genes and regulatory mechanism in AML’s development and progress need to be identified. Studies revealed that lncRNAs play important roles in diverse gene expression and cellular processes regulation. Dysregulation of lncRNAs has been reported to contribute to oncogenesis and tumor metastasis, including AML [22, 23].Therefore, the investigation of lncRNAs’ expression and function could help us to understand leukemogenesis and identify novel therapeutic targets. However, to date, only a few studies have reported mechanical and functional characterization of AML-associated aberrant gene networks [24–27].

Recently, many studies have reported that endogenous lncRNA had miRNA responsive elements (MRE) and modulated miRNAs via acting as miRNA sponges and binding with them [28]. These lncRNAs acting as competing endogenous RNAs (ceRNAs) participate in post-transcriptional regulation by interfering with the miRNA pathways [29]. ceRNAs have been shown to play critical roles in diverse biological functions and the disruption of the equilibrium of ceRNAs were implicated in tumorigenesis [3, 28]. For example, ATB lncRNA blocked miR-200 family by binding to its targets and upregulated ZEB1 and ZEB2, thereby inducing EMT and invasion in hepatocellular carcinoma [30]. Hence, understanding the intricate interplay among protein-coding messenger RNAs, miRNAs and lncRNA would help to identify gene regulatory networks which played critical roles in the progress and development of younger CN-AML. In the present study, we identified prognosis related specific lncRNAs, miRNAs and mRNAs of CN-AML from TCGA database. Moreover, we constructed a ceRNA network which could provide an integrated biological view based on the bioinformatics differential analysis. Some of these network have been reported to be solid tumor-associated gene-network, such as lncRNA–miRNA (CASC2-miR-221 [31]), miRNA–mRNA (miR-221-RAB1A [32] ,miR-25-PTEN [33], and miR-221-FMR1 [34]). The ceRNA network that we built revealed a previously unknown ceRNA regulatory network in pediatric and adolescent CN-AML. However, we failed to find target mRNAs when we constructed a ceRNA network with lncRNAs positively associated with overall survival and miRNAs negatively associated with prognosis.

In order to identify the function of the key lncRNAs, miRNAs and mRNAs in the ceRNA network, we analyzed their associations with OS by Kaplan–Meier curve. Our results suggested that 6 lncRNAs, 1 miRNA and 14 mRNAs were associated with CN-AML overall survival. Among them, has-mir-363 has been reported to be associated with prognosis of AML [35]; lncRNA CRNDE played critical roles in promoting cell proliferation, invasion and migration of solid tumor [36]; mRNA HMBOX1 and KIF5B was involved in the carcinogenesis [37, 38]. The other lncRNAs (AL356475.1, AC011498.1, AC092811.1, LINC00158 and LINC00504) and mRNAs (BRWD1, CNIH1, FMR1, GOLGA8J, GPBP1L1, NUFIP2, PTAR1, RRN3, SNX4, TMF1, ZFC3H1 and ZKSCAN8) were not reported previously. We analyzed the relationships of the above 35 key lncRNAs, 7 miRNAs with clinical features including age at diagnosis, gender, WBC at diagnosis, bone marrow blasts, peripheral blasts and molecular mutations (FLT3-ITD, NPM1, CEBPA). The results showed that AC011498.1, CRNED, LINC00504 and hsa-mir-363 were the indicators of CEBPA mutation of AML. Furthermore, AC011498.1 was also associated with NPM1 mutation. LncRNA CRNDE and miRNA hsa-mir-363 have been previously reported to be associated with clinical features of leukemia [35, 39]. However, the other lncRNAs we found here have not been reported to be the indicators of relevant features previously. The imprint of genetic ancestry and population structure carried in the genome of each individual and groups has led to the remarkable racial and ethnic diversity, which integrate biological, geopolitical, linguistic and cultural factors and are widely applied in population study [40]. Besides, Bonham et al. [41] have reported that the assessment of race and ethnicity at the individual level will make us closer to more individualized genetic-based medicine. Hence, we have estimated the association between miRNAs, race and ethnicity, but we failed to find any relation among them.

To date, the prognostic gene expression signatures have been extensively proposed for targeted therapies in cancer patients with significantly heterogeneous outcomes. However, the potential relationships of the genes have been ignored [13]. Genes were considered as independent individuals, which may result in undermining potential relationship between genes [13]. To overcome these challenges and to reduce the difficulty in biological interpretation, we constructed a weighted correlation network [14]. Then, we analyzed the relationships among the modules, OS, FAB category and the molecular mutations associated with prognosis (FLT3-ITD, NPM1, CEBPA). To identify biological pathways that were indicators of AML’s prognosis, significant modules associated with OS and clinical features were selected to carry out functional enrichment analysis. The GO analysis showed that the functions of significant differences in the aspects of “transcription, DNA-templated” (ontology: BP), “integral component of membrane” (ontology: CC) and “DNA binding” (ontology: MF). Furthermore, by KEGG pathway analysis, we identified 4 biological pathways associated with OS which may predict prognosis and guide treatment in younger CN-AML patients, including ‘Transcriptional misregulation in cancer, Regulation of actin cytoskeleton, TGF-beta signaling pathway and Ether lipid metabolism’. Recently, many studies have shown epigenetic abnormalities caused by ‘Transcriptional misregulation in cancer’ play important roles in tumor biology, such as DNA methylation, histone modifications and noncoding RNAs [42–44]. Therefore, we selected the most enriched network ‘Transcriptional misregulation in cancer’ to verify the relationship between epigenetic abnormalities and CN-AML patients’ prognosis. The result showed that the ‘Transcriptional misregulation in cancer’ pathway could strongly predict younger CN-AML patients’ survival. In our study, we only showed FLT3-ITD mutations could predict unfavorable outcome of pediatric CN-AML patients (P = 0.003), but we failed to find the relationship between NPM1 and CEBPA mutation and patients’ prognosis. One reason may be the sample size in our study was small and it couldn’t come to a significant conclusion. Another reason may be that patients simultaneously possess FLT3-ITD, NPM1 or CEBPA mutations in our study. Some reports have shown FLT3-ITD could implement a negative effect on OS irrespective of NPM1 or CEBPA mutations [45–47].

In PPI network analysis, we identified 30 hub genes and selected the top ten genes to analysis in detail. Among them, the PIK3CG gene, which encodes the catalytic subunit of phosphoinositide 3-OH-kinase-γ (PI3Kγ) namely p110γ, is located in chromosome band 7q22 and is often missing in myeloid malignancies [48]. Grimwade et al. [48] has implied PIK3CG was evaluated as a candidate suppressor gene of myeloid tumor. Dysregulation of apoptosis, which leads to the accumulation of tumor cells by slowing the rate of cell turnover, is a hallmark of cancer [49]. BCL-2 proteins encoded by BCL-2 gene can be classified into two families, anti-apoptotic and pro-apoptotic proteins. As a central regulator of cell polarity, loss of CDC42 suppresses AML cell polarity and division asymmetry [50]. Primary AML cells show constitutive release of a wide range of chemokines (including CCL5) involved in leucocyte chemotaxis, differentiation and angiogenesis [51]. LEP acting as a growth factor promotes cellular proliferation and may affect leukemic hematopoiesis [52]. Hence, the genes PIK3CG, BCL-2, CDC42, CCL5 and LEP in the treatment of AML are now being widely and actively taken into consideration. In previous studies, there were no reports showing the other top ten hub genes were associated with AML.

## Conclusion

Taken together, we filtered survival related specific lncRNAs, miRNAs and mRNAs after analyzing by univariate cox’s model from TCGA database. We successfully constructed a survival specific ceRNA network which could provide a new approach to lncRNA research in younger CN-AML. Importantly, we constructed a weighted correlation network to overcome the difficulty in biological interpretation of independently individual genes. We showed that 4 biological pathways associated with OS may be considered as potential specificity biomarkers in the diagnosis, prognosis and classification of pediatric and adolescent CN-AML. In addition, we select the most enriched network ‘Transcriptional misregulation in cancer’ to verify that it could accurately predict pediatric and adolescent CN-AML patients’ prognosis.

## Additional files

**Additional file 1: Figure S1.** Kaplan–Meier survival curves for 6 lncRNAs associated with overall survival from ceRNA network.

**Additional file 2: Figure S2.** Kaplan–Meier survival curves for 1 miRNA associated with overall survival from ceRNA network.

**Additional file 3: Figure S3.** Kaplan–Meier survival curves for 14 mRNAs associated with overall survival from ceRNA network.

**Additional file 4: Table S1.** The correlation among cancer specific lncRNAs, miRNAs and clinical features.

**Additional file 5: Table S3.** The correlation among cancer specific miRNAs, race and ethnicity.

**Additional file 6: Table S2.** Univariate Cox analysis of clinical parameters with the prognosis.

## Abbreviations

AML: acute myeloid leukemia
CN-AML: cytogenetically normal acute myeloid leukemia
TCGA: the cancer genome atlas
OS: overall survival
AUC: the area under receiver operating characteristic curve
LOO-CV: leave-one-out cross validation
GO: gene ontology
DAVID: The Database for Annotation, Visualization and Integrated Discovery
KEGG: Kyoto Encyclopedia of Genes and Genomes
PPI: the protein–protein interactions
CeRNA: competitive endogenous RNA
lncRNA: long noncoding RNA
miRNA: microRNA
mRNA: messenger RNA
