# Supplementary material for: Identification of long non-coding RNA competing interactions and biological pathways associated with prognosis in pediatric and adolescent cytogenetically normal acute myeloid leukemia
Source: Cancer Cell Int. 2018 Aug 28;18:122. doi: 10.1186/s12935-018-0621-0 (PMC6114287; doi:10.1186/s12935-018-0621-0)
Supplement: Supplementary file 4 — Additional file 4: Table S1. The correlation among cancer specific lncRNAs, miRNAs and clinical features. [file 12935_2018_621_MOESM4_ESM.docx]

Table S1. The correlation between cancer specific lncRNAs, miRNAs and clinical features

| **Comparisons** | ***lncRNAs*** | ***miRNAs*** |
| --- | --- | --- |
| WBC at diagnosis | CASC2，CRNDE |  |
| bone marrow blasts | CRNDE |  |
| peripheral blasts | CRNDE，LINC00504 | hsa-mir-363 |
| NPM1 mutation | AC011498.1 |  |
| CEBPA mutation | AC011498.1，CRNED，LINC00504 | hsa-mir-363 |
