# Supplementary material for: Identification of long non-coding RNA competing interactions and biological pathways associated with prognosis in pediatric and adolescent cytogenetically normal acute myeloid leukemia
Source: Cancer Cell Int. 2018 Aug 28;18:122. doi: 10.1186/s12935-018-0621-0 (PMC6114287; doi:10.1186/s12935-018-0621-0)
Supplement: Supplementary file 5 — Additional file 5: Table S3. The correlation among cancer specific miRNAs, race and ethnicity. [file 12935_2018_621_MOESM5_ESM.docx]

**Table S3. The correlation among cancer specific miRNAs, race and ethnicity.**

| Variants | hsa-mir-25 | | | hsa-mir-363 | | | hsa-mir-551a | | | | hsa-mir-221 | | | | hsa-mir-100 | | | |
| --- | --- | --- | --- | --- | --- | --- | --- | --- | --- | --- | --- | --- | --- | --- | --- | --- | --- | --- |
|  | **Low(14)** | **High(13)** | **P-value** | **Low(14)** | **High(13)** | **P-value** | **Low(14)** | **High(13)** | **P-value** | **Low(14)** | | **High(13)** | **P-value** | **Low(14)** | | **High(13)** | **P-value** |  |
| Ethnicity |  | | | | | | | | | | | | | | | | | |
| Hispanic or Latino | 2 | 4 | 0.3845 | 3 | 3 | >0.999 | 2 | 4 | 0.3845 | 4 | | 2 | 0.6483 | 5 | | 1 | 0.1647 |  |
| Not Hispanic or Latino | 12 | 8 | 0.2087 | 11 | 9 | 0.6776 | 11 | 9 | 0.6776 | 10 | | 10 | >0.999 | 8 | | 12 | 0.1602 |  |
| Unknown | 0 | 1 | 0.4815 | 0 | 1 | 0.4815 | 1 | 0 | >0.999 | 0 | | 1 | 0.4815 | 1 | | 0 | >0.999 |  |
| RACE |  | | | | | | | | | | | | | | | | | |
| American Indian or Alaska Native | 0 | 1 | 0.4815 | 1 | 0 | >0.999 | 0 | 1 | 0.4815 | 0 | | 1 | 0.4815 | 0 | | 1 | 0.4815 |  |
| Asian | 1 | 1 | >0.999 | 1 | 1 | >0.999 | 1 | 1 | >0.999 | 0 | | 2 | 0.2222 | 1 | | 1 | >0.999 |  |
| Black or African American | 2 | 1 | >0.999 | 2 | 1 | >0.999 | 3 | 0 | 0.2222 | 2 | | 1 | >0.999 | 1 | | 2 | 0.5956 |  |
| Native Hawaiian or other Pacific Islander | 1 | 1 | >0.999 | 1 | 1 | >0.999 | 2 | 0 | 0.4815 | 2 | | 0 | 0.4815 | 1 | | 1 | >0.999 |  |
| White | 9 | 8 | >0.999 | 7 | 10 | 0.2365 | 8 | 9 | 0.6946 | 9 | | 8 | >0.999 | 10 | | 7 | 0.4401 |  |
| Unknown | 1 | 1 | >0.999 | 2 | 0 | 0.4815 | 0 | 2 | 0.2222 | 1 | | 1 | >0.999 | 1 | | 1 | >0.999 |  |
