# Supplementary material for: Identification of long non-coding RNA competing interactions and biological pathways associated with prognosis in pediatric and adolescent cytogenetically normal acute myeloid leukemia
Source: Cancer Cell Int. 2018 Aug 28;18:122. doi: 10.1186/s12935-018-0621-0 (PMC6114287; doi:10.1186/s12935-018-0621-0)
Supplement: Supplementary file 6 — Additional file 6: Table S2. Univariate Cox analysis of clinical parameters with the prognosis. [file 12935_2018_621_MOESM6_ESM.docx]

Table S2: Univariate Cox analysis of clinical parameters with the prognosis

| **Variants** | ***P*-value** |
| --- | --- |
| Age at diagnosis | 0.042 |
| Gender | 0.120 |
| Bone marrow blasts | 0.694 |
| Peripheral blasts | 0.530 |
| FLT3-ITD positive | 0.003 |
| NPM1 mutation | 0.384 |
| CEBPA mutation | 0.081 |
| WT1 mutation | 0.039 |
